# *Arg399Gln XRCC1* Polymorphism and Risk of Squamous Cell Carcinoma of the Head and Neck in Jordanian Patients

**DOI:** 10.31557/APJCP.2020.21.3.663

**Published:** 2020-03

**Authors:** Ammar Sobiahe, Eman Hijazi, Hamzeh J. Al-Ameer, Yazan Almasri, Yazun Jarrar, Malek Zihlif, Maha Shomaf, Baeth Al-Rawashdeh

**Affiliations:** 1 *Department of Dental Surgery, *; 2 *Department of Pathology, Islamic Hospital,*; 4 *Department of Pharmacology, Faculty of Medicine, *; 6 *Department of Pathology, *; 6 *Department of Pathology, *; 7 *Department of Otolaryngology and Head and Neck Surgery, The University of Jordan, *; 5 *Department of Pharmacy, Faculty of Pharmacy, Al-Zaytoonah University of Jordan, Amman, *; 3 *Department of Biology and Biotechnology, American University of Madaba, Madaba, Jordan. *

**Keywords:** XRCC1, squamous cell carcinoma, head and neck, Jordanians

## Abstract

**Background and Objective:**

X-ray repair cross-complementing group1 (XRCC1) is a key protein in base excision repair and closely associated with the coordination of the base excision repair pathway. Many studies have focused on *XRCC1 SNPs* and have shown an associated between these SNPs and the risk of several types of cancers, including head and neck cancer. There are many single nucleotide polymorphisms *XRCC1* gene (SNPs) and the most common SNP that result in amino acid substitutions is exon 10 (Arg399Gln). This study aimed to investigate the association between *Arg399Gln SNP* and the risk of squamous cell carcinoma of the head and neck.

**Materials and Methods:**

Ninety nine patients with squamous cell carcinomas of the head and neck and 89 healthy adult controls were enrolled in this study. The *Arg399Gln* in *XRCC1* allele was genotyped using polymerase chain reaction-restriction fragment length polymorphism method.

**Results:**

In the single-locus analyses, *Arg399Gln SNP* showed a significant association with head and neck cancer risk (p value = 0.016 and odd ratio of 1.8). On the genotype level, we applied three analysis models, namely co-dominant, dominant, and recessive genotypes. Arg/Arg homozygous major genotype was significantly (p value <0.05) associated with head and neck squamous cell carcinoma incidence with odd ratio of 2.23 and 2.24 for the co-dominant and recessive models, respectively.

**Conclusion:**

The findings indicated that *Arg399Gln* allele was associated with squamous cell carcinoma of the head and neck among Jordanian patients. This allele might be used as a genetic biomarker of squamous cell carcinoma of the head and neck.

## Introduction

DNA damage caused by UV, ionizing radiation, or environmental chemical agents can initiate human cancer (Munter, 2011). However, human body has a set of complex DNA repair systems safeguarding the integrity of the genome (Brambilla, 2010; Munter, 2011). Base excision repair (BER) is considered to be the primary guardian against damage that results from cellular metabolism, including reactive oxygen species, methylation, deamination, and hydroxylation (de Laat et al., 1999). Many studies have illustrated that the genetic variants in BER cause biochemical alterations that are associated with risk of various cancers (de Laat et al., 1999).

X-ray repair cross-complementing group 1 (XRCC1) is a key protein in BER and closely associated with BER pathway coordination due to interacting with most components of the BER short-patch pathway (Caldecott et al., 1996; Marintchev et al., 2000). There are many single nucleotide polymorphisms of *XRCC1* gene (SNPs), some are located in the promoter region of the gene and others are located in the exons (Caldecott et al., 1996; Marintchev et al., 2000). The most common SNPs that result in amino acid substitutions are in exon 6 (Arg194Trp), exon 9 (Arg280His), and exon 10 (Arg399Gln). Interestingly, these amino acid alterations may affect the protein-protein interaction between XRCC1 and other BER proteins, which in turn may alter DNA repair capability (Shen et al., 1998). 

Many studies have focused on *XRCC1 Arg399Gln SNP* and have shown its association with the risk of several types of cancers, including head and neck cancer (Caldecott et al., 1996; de Laat et al., 1999). Interestingly, a meta-analysis that focused on the association between *Arg399Gln* and head and neck cancer recommended further studies to assess *Arg399Gln SNPs* in *XRCC1* as risk factors for developing head and neck cancer (Olshan et al., 2002). This study aimed to investigate the association between *Arg399Gln SNP* and the risk of squamous cell carcinoma of the head and neck. 

## Materials and Methods

*Study design*

A retrospective observational control study was conducted on 99 head and neck cancer patients and 89 healthy adult as controls. Patients were diagnosed with head and neck cancer at Jordanian Islamic hospital between 2004 and 2012. After being approved by the institutional review board (IRB) of Jordanian Islamic hospital, archived tissue samples taken from the tumors were used for DNA extraction inthisnstudy.

The statistical population of this study was selected among Jordanian referred to Islamic hospital. However, Jordanian generally are mixed population with different ethnic backgrounds. Generally speaking, Jordanians are nearly Caucasian.

The control group samples were also paraffin sample with non cancerous diagnoses. The diagnosis was branchial cyst and thyroglossal duct cyst. 

*DNA extraction and genotyping:*

The DNA was extracted from archived formalin-fixed paraffin-embedded tissue blocks using QIAamp DNA FFPE Tissue Kit (Qiagen, Germany). The extraction was done according to the manufacturer’s instructions. Polymerase chain reaction (PCR) –restriction fragment length polymerase (RFLP) assay was used to detect XRCC1 Arg39Gln polymorphisms. PCR reaction was performed using forward and reverse primers: 5` CAAGTACAGCCAGGTCCTAG-3` and 5` CCTTCCCTCATCTGGAGTAC -3’. Each run of PCR included a negative control lacking genomic DNA. 

Briefly, the PCR mixture (25 µl) was prepared using 200 ng of genomic DNA, 10 µl of Taq polymerase buffer, 0.2mM of dNTPs, 3.0 µl of MgCl2, 0.2 pM from each of the forward and reverse primers (Invitrogen, Carlsbad, CA, USA) and 2.5 U of Taq DNA polymerase. After initial denaturation at 95ºC for 3 min, 40 cycles were performed consisting of denaturation step at 95ºC for 30 sec, annealing step at 59ºC for 30 sec, elongation step at 72 ºC for 30 sec, and a final elongation step at 72ºC for 5 min. The resulting PCR products were separated and detected in ethidium bromide-containing 2.0% agarose gel.

*Restriction enzyme analysis*

The PCR products were digested with NciI enzyme according to the manufacturer’s instructions. DNA fragments were separated on 3% agarose and visualized by ethidium bromide. Analysis of genotypes was done using restriction enzymes, which recognized specific sequence in the DNA and digested at that sequence to give DNA fragments of precisely defined length.

*Statistical analysis*

Statistical analyses were performed using Statistical Package for Social Sciences (SPSS Inc., Chicago, Illinois) version 17.0. Continuous variables were analyzed using ANOVA. Odds ratios (OR) were calculated using the WEB odds ratio calculator at http://www.medcalc.org/calc/odds_ratio.php. P-value of < 0.05 was considered significant. The differences between alleles frequencies in different populations were evaluated by the Z test using the online z test calculator at http://www.socscistatistics.com/tests/ztest/Default2.aspx.

## Results

The 99 HNSCC cases including 34 females (0.34) and 65 males (66 %) were recruited as the experimental group in this study. The median age of the patients was 60.42 ± 15.47 years old. In the control group, 89 participants with median age of 43.6 ± 20.2 were included. In the latter, there were 40 females (45%) and 59 males (55 %) (Table1). 

In the single-locus analyses, Arg399Gln SNP showed a significant association with head and neck cancer risk (P = 0.016). Interestingly, the frequency of Arg allele was higher in the cancer patients, indicating that the Arg allele may have a risk effect with an odds ratio of 1.8. On the genotype level, three analysis models were applied, namely co-dominant, dominant, and recessive genotypes. Arg/Arg homozygous major genotype was found to be associated with head and neck cancer incidence in the co-dominant model (odds ratio of 2.23). This association was also evident in the recessive model with a p-value of 0.007and an odds ratio of 2.24.

**Table 1:** Genotyping Frequencies and Allelic Distribution

SNP rs25487(n=188) Arg399Gln	Control (n=89)	SCCHN (n=99)	P-value	Odds Ratio (95% CI)
Arg/Arg (n=110)	43 48.30%	67 67.70%	0.027	reference
Arg/Glu (73)	43 48.30%	30 30.30%		2.23 (1.2-4.1)
Gln/Gln (n=5)	3 3.40%	2 2.00%		2.34(0.34-14.6)
Arg/Arg +Arg/Glu (n=183)	86 96.60%	97 98.00%	0.566	0.59 (0.1-3.6)
Gln/Glu (n=5)	3 3.40%	2 2.00%		reference
Glu/Glu +Arg/Gln (n=78)	46 (36.90%)	32 (41.1)	0.007	2.24 (1.24-4)
Arg/Arg	43 (52.10%)	67 (57.90%)		reference
Alleles				
Gln	49 (28%)	34 (17%)	0.016	1.8 (1.12-3)
Arg	129 (72%)	164 (83%)		reference

## Discussion

In this study, we investigated the association between the most common polymorphism on *XRCC1* gene, *Arg399Gln*, and the risk of head and neck cancer. The frequency of Arg allele was significantly (p = 0.016) higher in the cancer patients than in the control group, indicating that the Arg allele may have a risk effect with an odds ratio of 1.8. Interestingly, *Arg399Gln* polymorphism frequency in the control group was in agreement with the reported one in Caucasian populations (Skjelbred et al., 2006). On the genotype level, Arg/Arg homozygous major genotype was found to be associated with head and neck cancer incidence in the co-dominant model (odds ratio of 2.23). This association was also evident in the recessive model with a p-value of 0.007 and an odds ratio of 2.24. 

The observed protective effect of the codon 399Gln allele in our study was also found in several previous studies on association between cancer and *XRCC1 *genotype.* XRCC1* genotype has been reported to be a protective genetic biomarker among bladder cancer (Stern et al., 2001), pancreatic adenocarcinoma (Duell et al., 2002), and gastric cancer patients (Ratnasinghe et al., 2004). The reduced cancer risk found in our study, as well as other studies, was congruent with the hypothesis that the variant XRCC1 protein and its diminished DNA repair could enhance damage-related apoptosis in individual cells. This hypothesis was suggested by Seedhouse et al. who investigated the association between *XRCC1 *polymorphisms and secondary (treatment-related) leukemia and found a similar inverse association between cancer risk and *XRCC1* polymorphism (Seedhouse et al., 2002). On the other hand, some reports indicated that the *Arg399Gln* genotype increased the risk of HNSCC (Tae et al., 2004). Previous studies showed that the variant allele increased the accumulation of DNA damage, leading to increased incidence of malignancy (Tae et al., 2004). 

One limitation of this study was related to the relatively small number of cases and controls. More extensive studies with larger sample sizes are needed to validate the genetic effects of XRCC1 polymorphisms on head and neck squamous cell carcinoma.

In conclusion, the findings of this study indicated that the Arg399Gln allele was a possible genetic biomarker of head and neck cancer risk in Jordanian patients. Further studies are needed to validate this allele as a biomarker of head and neck cancer as a diagnostic and predictive marker.
